# Creating a Vaccine-like Supplement against Respiratory Infection Using Recombinant *Bacillus subtilis* Spores Expressing SARS-CoV-2 Spike Protein with Natural Products

**DOI:** 10.3390/molecules28134996

**Published:** 2023-06-26

**Authors:** Ben Chung-Lap Chan, Peiting Li, Miranda Sin-Man Tsang, Johnny Chun-Chau Sung, Keith Wai-Yeung Kwong, Tao Zheng, Sharon Sze-Man Hon, Ching-Po Lau, Wen Cheng, Fang Chen, Clara Bik-San Lau, Ping-Chung Leung, Chun-Kwok Wong

**Affiliations:** 1 Institute of Chinese Medicine and State Key Laboratory of Research on Bioactivities and Clinical Applications of Medicinal Plants, The Chinese University of Hong Kong, Hong Kong, China; benchan99@cuhk.edu.hk (B.C.-L.C.); peiting@link.cuhk.edu.hk (P.L.); miranda.tsang@rmit.edu.au (M.S.-M.T.); zhengtao@cuhk.edu.hk (T.Z.); sharonhon@link.cuhk.edu.hk (S.S.-M.H.); chingpolau@cuhk.edu.hk (C.-P.L.); wencheng@cuhk.edu.hk (W.C.); fangchen@cuhk.edu.hk (F.C.); claralau@cuhk.edu.hk (C.B.-S.L.); pingcleung@cuhk.edu.hk (P.-C.L.); 2 China-Australia International Research Centre for Chinese Medicine, School of Health and Biomedical Sciences, STEM College, RMIT University, Bundoora, VIC 3083, Australia; 3 Research Department, DreamTec Cytokines Limited, Hong Kong, China; johnnysung@dreamtec.hk (J.C.-C.S.); keithkwong@dreamtec.hk (K.W.-Y.K.); 4 Department of Chemical Pathology, The Chinese University of Hong Kong, Prince of Wales Hospital, Shatin, NT, Hong Kong, China; 5 Li Dak Sum Yip Yio Chin R & D Centre for Chinese Medicine, The Chinese University of Hong Kong, Hong Kong, China

**Keywords:** *Bacillus subtilis*, cytokines, dendritic cells, macrophages, SARS-CoV-2, *Astragalus membranaceus* (Fisch.) Bge (AM), *Coriolus versicolor* (CV)

## Abstract

Vaccination is the most effective method of combating COVID-19 infection, but people with a psychological fear of needles and side effects are hesitant to receive the current vaccination, and alternative delivery methods may help. *Bacillus subtilis*, a harmless intestinal commensal, has recently earned a strong reputation as a vaccine production host and delivery vector, with advantages such as low cost, safety for human consumption, and straightforward oral administration. In this study, we have succeeded generating “S spores” by engineering *B. subtilis* with spore coat proteins resembling the spike (S) protein of the ancestral SARS-CoV-2 coronavirus. With the addition of two immunostimulating natural products as adjuvants, namely *Astragalus membranaceus* (Fisch.) Bge (AM) and *Coriolus versicolor* (CV), oral administration of S spores could elicit mild immune responses against COVID-19 infection without toxicity. Mucosal IgA against the S protein was enhanced by co-feeding with AM and CV in an S spores-inoculated mouse model. Faster and stronger IgG responses against the S protein were observed when the mice were fed with S spores prior to vaccination with the commercial COVID-19 vaccine CoronaVac. In vitro studies demonstrated that AM, CV, and *B. subtilis* spores could dose-dependently activate both macrophages and dendritic cells by secreting innate immunity-related IL-1β, IL-6, and TNF-α, and some other proinflammatory chemokines and cytokines. In conclusion, the combination of S spores with AM and CV may be helpful in developing a vaccine-like supplement against respiratory infection.

## 1. Introduction

The Coronavirus Disease 2019 (COVID-19) pandemic has been ravaging the world for more than three years. Despite some vaccines having been developed and applied to combat and prevent the disease, COVID-19 continues to rage and remains unpredictable due to the emergence of the Omicron variant and its descendant subvariants with increasing infectivity [1,2,3,4]. The BQ and XBB subvariants of severe acute respiratory syndrome coronavirus 2 (SARS-CoV-2) Omicron are now spreading rapidly, possibly due to spike mutations that alter the antibody required by the host, resulting in immune escape [5]. Interventions are urgently needed to prevent the new emerging variant and/or to treat the disease. Genetic engineering of the spores of *Bacillus subtilis* (*B. subtilis*) has raised interest in vaccine development. The *B. subtilis* spores are desirable as a carrier for vaccines because the FDA generally recognizes them as safe, and the spores can act as an adjuvant to boost immune response, especially innate immunity [6,7]. *B. subtilis* has been studied for decades and developed as a bioactive supplement for immunomodulation [8]. Specific proteins with known targets for the induction of adaptive immune responses can be used as antigens for vaccine development. The ease of administration is another major advantage for the development of oral vaccination against infectious diseases. We have successfully generated *B. subtilis* spores expressing the SARS-CoV-2 receptor binding domain (RBD) of the spike (S) protein genetically fused to the surface-exposed spore coat proteins CotC of the *B. subtilis* spores [9], and a similar *B. subtilis* construct has recently been produced by another group [10].

To further enhance the immunoreactivity of *B. subtilis* in vaccine development, an additional agent may be essential, and beta (β)-glucan enriched natural products may be a good choice. In our earlier study using a murine subcutaneous immunization model with Globo H and GD3 carbohydrates conjugated to keyhole limpet hemocyanin (KLH) as an immunogen mixed with a panel of β-glucan-enriched natural products as immunological adjuvants, we found that *Astragalus membranaceus* (Fisch.) Bge (AM) and *Coriolus versicolor* (CV) are the most active immune stimulants with adjuvant activity [11,12,13]. We found that the bioactive polysaccharides isolated from AM and CV exhibited significant immunomodulatory effects by stimulating the proliferation of human peripheral blood mononuclear cells (PBMC) and enhancing innate immunity-related IL-1β and TNF-α production [14,15,16,17]. The immunostimulatory properties of AM and CV polysaccharides may promote trained immunity [18] with a long-term enhancement of innate immune responses and induce heterologous protection against infection. The objectives of this study were to investigate the immunopotentiating effects of *B. subtilis* spores, AM and CV, by examining the (i) in vitro innate immunity-boosting effects using human macrophages and intestinal HT-29 cells co-culture and monocyte-derived dendritic cells (DC), (ii) the in vivo oral vaccination-like activities using a mouse immunization model [19,20], and (iii) the use of *B. subtilis* spores, S spores, AM, and CV as an oral supplement for CoronaVac vaccination.

## 2. Results

### 2.1. MTT Cytotoxicity Test

**Figure 1:**
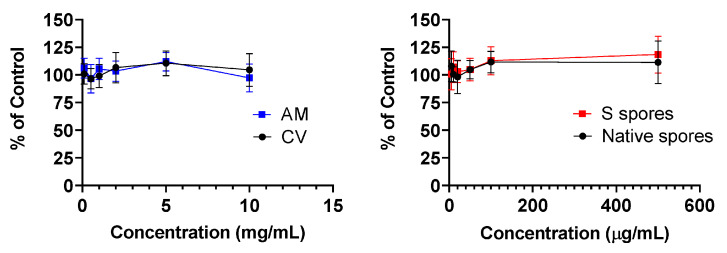
Cellular toxicity (MTT assay) of *Astragalus membranaceus* (Fisch.) Bge (AM), *Coriolus versicolor* (CV), native *B. subtilis* spores (Native spores), or genetically engineered *B. subtilis* (S spores) on human peripheral blood mononuclear cells (PBMC) (n = 4).

No cytotoxicity was observed in human PBMC treated with AM, CV (0.1 to 10 mg/mL), native, and S protein *B. subtilis* spores (5 to 500 μg/mL) for 48 h (Figure 1).

### 2.2. Co-Culture of Human Monocyte-Derived Macrophages with Intestine HT-29 Cells and Dendritic Cells (DC)

**Figure 2:**
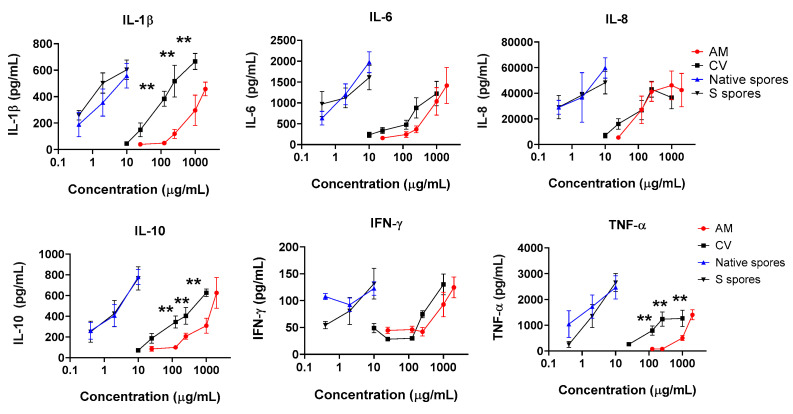
The immunostimulating effects of *Astragalus membranaceus* (Fisch.) Bge (AM), *Coriolus versicolor* (CV), native *B. subtilis* spores (native spores) or genetically engineered *B. subtilis* (S spores) on the production of proinflammatory cytokines (IL-1β, IL-6, IL-8, IL-10, IFN-γ, and TNF-α) from human macrophages and HT-29 cells co-culture (n = 4). ** *p* < 0.01 comparing to AM group.

Human intestinal epithelial HT-29 cells were cultured to confluence, then human monocyte-derived macrophage suspensions (5 × 10^5^/mL) were applied to HT-29 cells in the culture plate. The epithelial cells–monocyte co-cultures were incubated with or without native spores, S spores, AM, and CV, for a further 18 h. The concentrations of proinflammatory cytokines (IL-1β, IL-6, IL-8, IL-12, IFN-γ, and TNF-α) of innate immunity in the culture supernatant were quantitated (Figure 2). The production of IL-1β, IL-6, IL-8, IL-10, IFN-γ, and TNF-α, was dose-dependently increased when cells were incubated with AM, CV, or spores (Figure 2). The stimulatory effects of *B. subtilis* spores were more potent than AM and CV. CV (100–1000 μg/mL) produced significantly higher concentrations of IL-1β, IL-10, and TNF-α when compared to AM. Compared with macrophages and HT-29 culture separately without treatment, the cytokine production profiles were similar to the co-culture of macrophages with HT-29 cells (Figure A5). Results can, therefore, exclude the possibility of the effect of HT29 cells on macrophages in co-culture.

**Figure 3:**
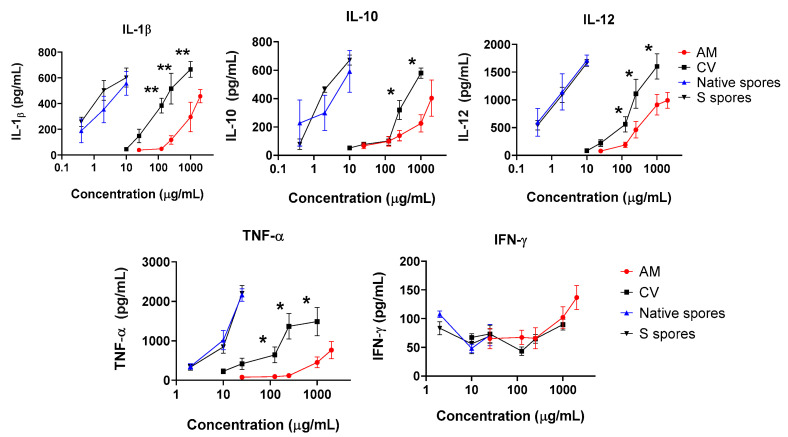
The immunostimulating effects of *Astragalus membranaceus* (Fisch.) Bge (AM), *Coriolus versicolor* (CV), native *B. subtilis* spores (native spores), or genetically engineered *B. subtilis* (S spores) on proinflammatory cytokines (IL-1β, IL-10, IL-12, IFN-γ, and TNF-α) productions from human monocyte-derived dendritic cells (DC) (n = 4). * *p* < 0.05 and ** *p* < 0.01 comparing with AM group.

IL-12 is one of the major cytokines secreted by activated DC. A significant and dose-dependent increase in IL-12, IL-1β, IL-10, and TNF-α was observed when cells were incubated with AM, CV, or spores. A small increase in anti-viral IFN-γ production was observed when a high dose of AM was used (2 mg/mL) (Figure 3). Similar to the co-culture of macrophage and HT-29 cells, the stimulatory effects of *B. subtilis* spores were more potent than AM and CV. CV (100–1000 μg/mL) produced significantly higher concentrations of IL-1β, IL-10, IL-12, and TNF-α when compared to AM. Taken together, *B. subtilis* or AM/CV could promote the immune response and has an immune-adjuvant effect on macrophages and DC, which are the principal antigen-presenting cells to B cells for antibody production in humoral immunity.

### 2.3. Oral Vaccination of B. subtilis Spores, AM, and CV

**Figure 4:**
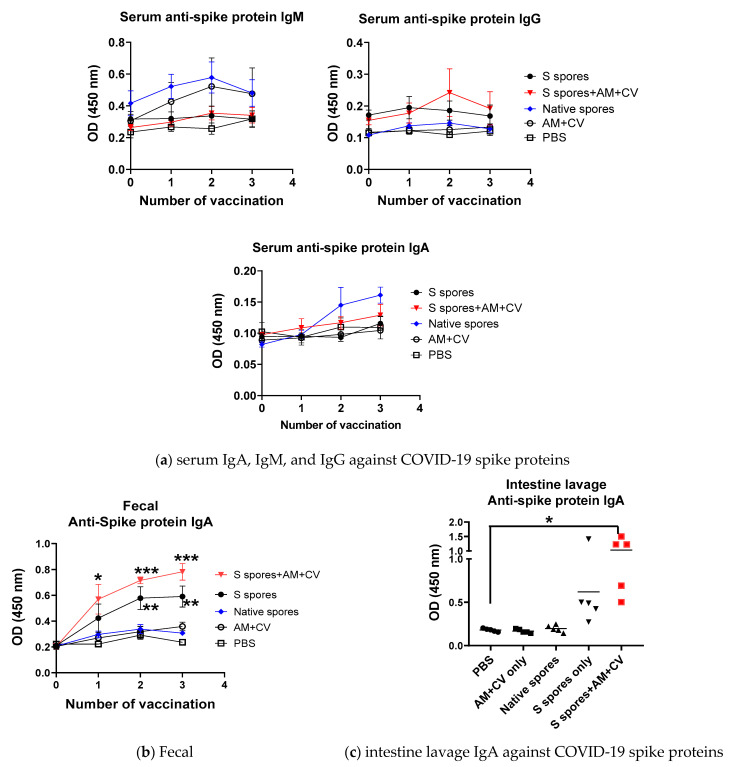
Summary of the pilot animal study. (**a**) Serum IgM, IgG, and IgA against SARS-CoV-2 spike protein from mice (n = 5) orally administrated with *Astragalus membranaceus* (Fisch.) Bge (AM), *Coriolus versicolor* (CV), native *B. subtilis* spores (native spores), or genetically engineered *B. subtilis* (S spores) or PBS (control) for three courses (days 1–4, days 14–17, and days 28–31). The presence of specific IgA against SARS-CoV-2 Spike protein in (**b**) feces and (**c**) intestinal lavage. * *p* < 0.05; ** *p* < 0.01 and *** *p* < 0.001 compared to control groups.

Mice were well-tolerated to the *B. subtilis* or AM/CV after 3 feeding sessions. Clinical changes (body weight, hair loss, body temperature) and eating habits (food and water intake, diarrhea) were not significantly affected by spores and AM/CV treatment. After the first oral treatment with spores and AM/CV, a relatively higher serum antibody IgM titer against the SARS-CoV-2 S protein was detected in mice that received native spores and AM/CV when compared with the mice fed with S spores with or without co-treatment with AM/CV (Figure 4a). After oral vaccination 2 times, a small increase in serum IgG titer against the SARS-CoV-2 S protein was observed in mice receiving S protein-engineered *B. subtilis* together with AM/CV. Very small increases in IgA production (OD 0.05) against the SARS-CoV-2 S protein were detected in the sera of mice from all groups. Adjuvant activities on the serum antibody production were not observed in the groups receiving S spores and co-treated with AM/CV. From the feces collected from different time points (Figure 4b), significantly higher levels of IgA against S protein were observed from mice fed with S protein-engineered *B. subtilis* alone, co-treatment with AM/CV compared with PBS group and mice fed only with native spores or AM/CV after first oral vaccination (all *p* < 0.05). For intestine lavage of the sacrificed mice (Figure 4c), a stronger IgA secretion against spike protein was observed in the mice from the S spores treatment group when compared with the PBS and AM/CV treatment-only groups, and one mouse produced a strong IgA response against SARS-CoV-2 S protein (OD = 1.4). For the S spores and AM/CV co-treatment group, an enhanced effect on IgA production was observed in all 5 mice (OD ranged from 0.4–1.5). A small but detectable IgM response against the S protein (not statistically significant) was observed in the mice after the first and second oral vaccination with the native or S spores. This may be due to the cross-reactivity between the surface antigens of the spores and the ancestral SARS-CoV-2 S protein.

**Figure 5:**
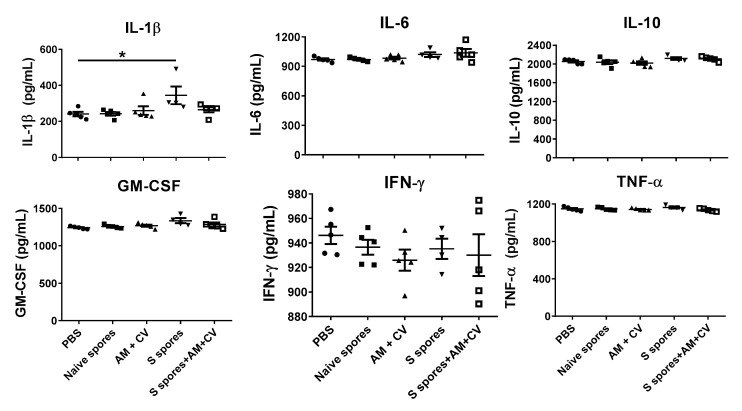
Serum cytokine profile of mice (n = 5) orally treated with *Astragalus membranaceus* (Fisch.) Bge (AM), *Coriolus versicolor* (CV), native *B. subtilis* spores (native spores), or genetically engineered *B. subtilis* (S spores) or PBS (control) for three courses (days 1–4, days 14–17, and days 28–31). * *p* < 0.05.

The serum cytokine profiles of the mice (IL-1β, IL6, IL-10, GM-CSF, IFN-γ, and TNF-α) for the different treatment groups are shown in Figure 5. In the S spores group, the serum concentration of IL-1β in one mouse was 490 pg/mL, which was 2-fold higher than the PBS group. Overall, a significant increase in IL-1β production was observed in the group of mice treated with S spores only compared to the control group. The increase in cytokine secretion was not observed in the mice treated with S spores and AM/CV.

### 2.4. Toxicity Studies

**Figure 6:**
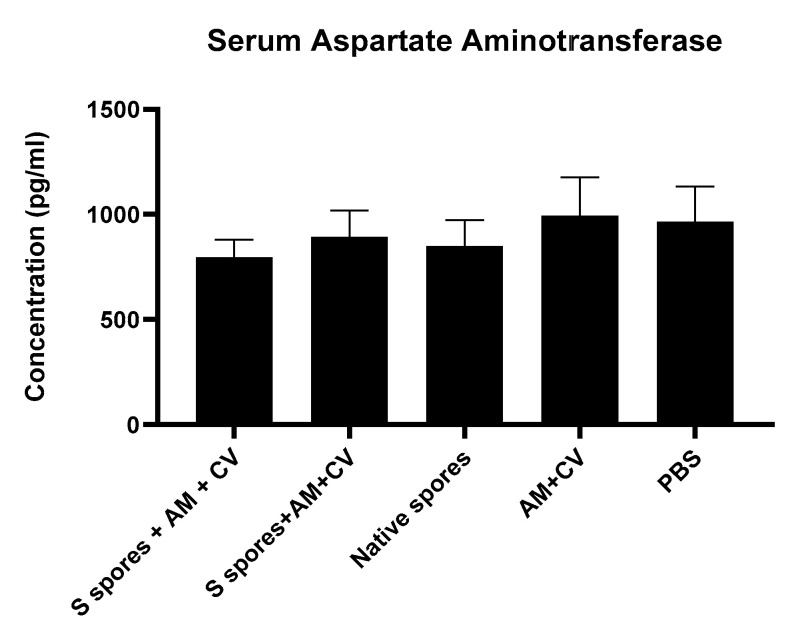
The concentrations of serum aspartate aminotransferase (AST) from mice treated with orally administrated *Astragalus membranaceus* (Fisch.) Bge (AM), *Coriolus versicolor* (CV), native *B. subtilis* spores (native spores), or genetically engineered *B. subtilis* (S spores) or PBS (control) for three courses (days 1–4, days 14–17, and days 28–31). Results were interpolated from the AST standard curves and corrected for sample dilution. The interpolated values corrected for dilution factor were plotted (mean +/− SEM, n = 5).

Biochemical markers (serum concentrations of the liver enzyme aspartate aminotransferase (AST)) in mice from different groups were measured to determine whether there was any liver function impairment caused by *B. subtilis* spores, AM, and CV (Figure 6) compared to the PBS group. There was no significant difference in the serum concentrations of liver AST in all treatment groups. It was concluded that the liver functions of the mice were not affected by oral administration of *B. subtilis* spores, AM, or CV.

**Figure 7:**
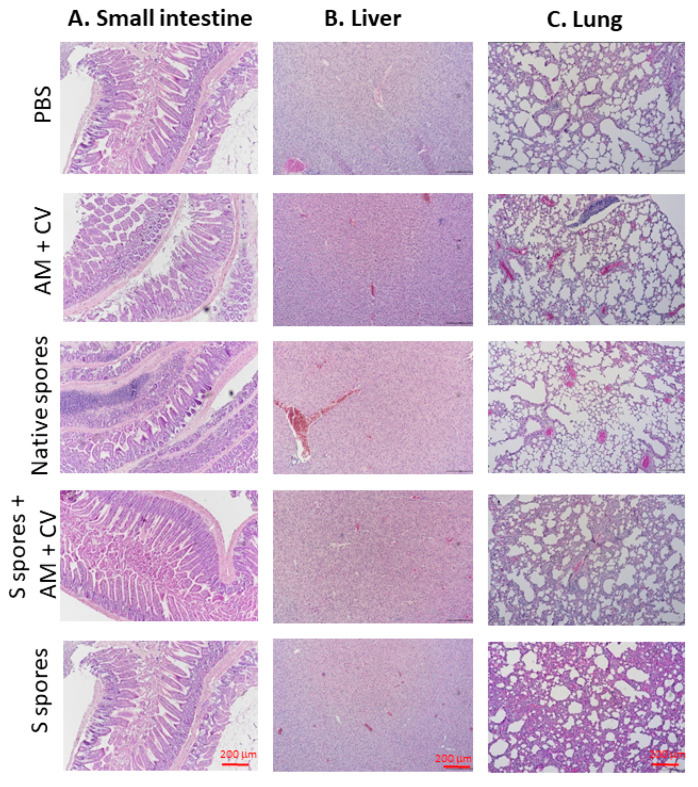
Representative hematoxylin and eosin (H & E) staining of (**A**) small intestine, (**B**) liver, and (**C**) lung at the terminal stage (magnification: 100×) of mice treated with native *B. subtilis* spores, S spores, AM and CV, or PBS (control) after three courses (days 1–4, days 14–17, and days 28–31).

The histopathological changes of (a) the small intestine, (b) the liver, and (c) the lung at the terminal stage are summarized in Figure 7. Compared with the non-treated group, inflammatory cell infiltrates, epithelial changes, disrupted epithelial barrier, and alterations in the overall mucosal architecture, presence of ulcerations, granulation tissue, irregular crypts, or crypt loss were not observed in the small intestine of any of the treatment groups. In the liver, hepatocellular morphology was similar in all treatment groups compared with the untreated group. Inflammation, hemorrhage, and architectural disruption of the liver were not observed. In lung tissue sections, the surrounding lung parenchyma was similar in all groups. Only mild infiltration of immune cells was observed in the groups treated with S spores and S spores + AM + CV when compared with the control group, but no bronchial epithelial damage, tissue necrosis, hemorrhage, or lesions were observed in these two treatment groups.

### 2.5. Adjuvant Effects of B. subtilis Spores, AM/CV on CoronaVac Vaccine

**Figure 8:**
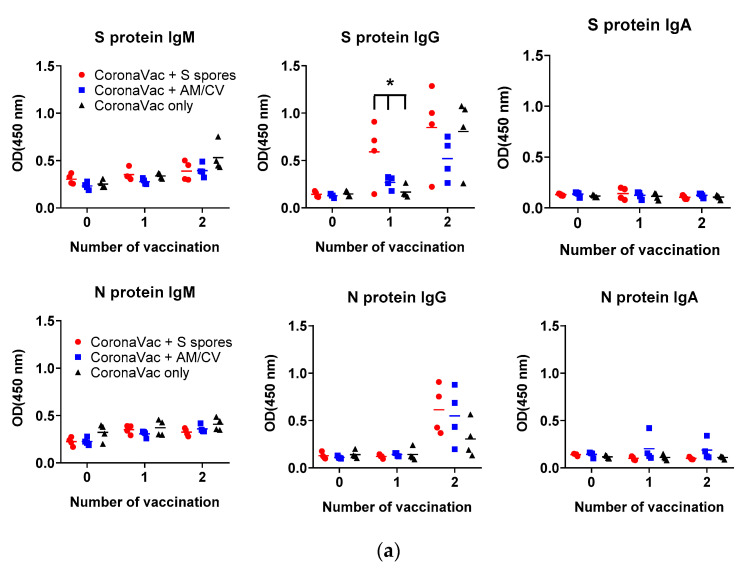

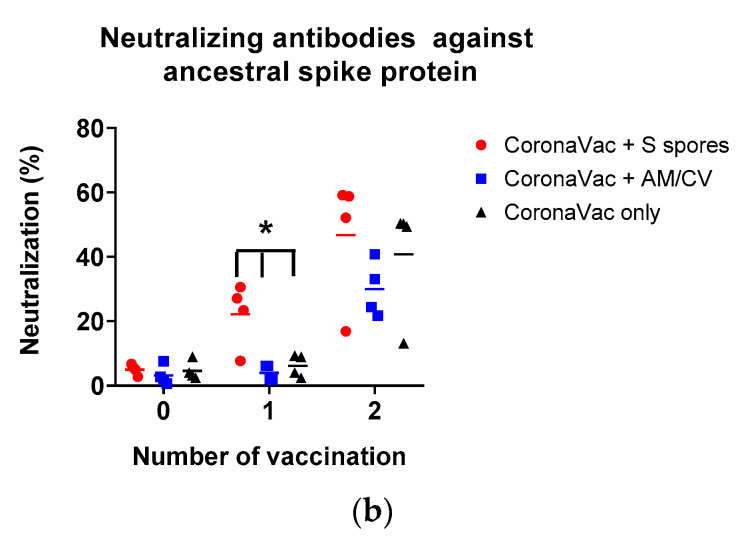
Summary of the CoronaVac animal study. (**a**) Serum (1:50) IgM, IgG, and IgA and (**b**) serum-neutralizing antibodies against ancestral SARS-CoV-2 spike protein from mice (n = 4) orally administered with *Astragalus membranaceus* (Fisch.) Bge (AM) and *Coriolus versicolor* (CV), genetically engineered *B. subtilis* (S spores), or PBS (control) for 2 courses (days 1–4 and days 14–17). The % neutralization of the positive control serum was 62.8 ± 0.3%. * *p* < 0.05.

The antibody production (IgA, IgM, and IgG against SARS-CoV-2 spike (S) and nucleocapsid (N) proteins) in the serum of all tested groups are shown in Figure 8. The antibody levels (IgA, IgM, and IgG against SARS-CoV-2 S and N proteins) of all groups tested were low on Day 1. After the first CoronaVac vaccination, a slight increase in IgM against both N and S proteins was observed in all vaccinated groups with no significant difference (all *p* > 0.05). For IgG antibodies against the N protein, the activities were low compared to the sera IgG before vaccination. A significant increase in IgG production against the S protein was observed only in the S spores-fed group (*p* < 0.05), suggesting that oral administration of S spores 14 days before CoronaVac could enhance the production of antibodies against the S protein of SARS-CoV-2. In the mice fed with AM and CV, IgM and IgG antibody activities against both N and S proteins were similar to those in the group that received CoronaVac alone. Notably, one mouse in the AM/CV group produced a relatively higher level of IgA against N protein after the first and second CoronaVac vaccinations. After the second CoronaVac vaccination, higher concentrations of IgG against S protein were observed in all vaccinated groups compared to the serum activities of the corresponding groups after the first vaccination. For N protein, higher levels of IgG against N protein were observed in both the S spores and AM/CV groups compared to the CoronaVac alone group. The mice sera were further tested for neutralizing antibody activities against SARS-CoV-2 using a commercially available SARS-CoV-2 neutralizing antibody ELISA assay (Figure 8b). After the first vaccination of CoronaVac, a significantly stronger neutralization activity was observed in the S spores-fed group (% neutralization = 22.2%) (*p* < 0.05) when compared with the other two groups: CoronaVac +AM/CV and CoronaVac only (4% and 6.2%). In summary, a significantly higher concentration of neutralizing IgG against S protein could be induced in mice with CoronaVac vaccination with prior oral administration of S spores.

## 3. Discussion

In SARS-CoV-2 infection, innate immunity-related monocytes and macrophages are also responsible for early pathogen recognition, initiation and resolution of inflammation, and repair of tissue damage [21]. Previous findings in the field of immune memory have shown that B and T cell-mediated adaptive immunity after infection is enhanced by “Trained Immunity” [22]. This effect has been investigated for the tuberculosis vaccine strain *Bacillus Calmette-Guerin* (BCG) [23]. Natea developed the concept of “Trained Immunity” based on epidemiological observations that people who have received commonplace vaccinations, such as BCG against *Mycobacterium tuberculosis* infection, tend to be more resistant to other infections. Using severe combined immunodeficiency (SCID) mice infected with *Candida albicans*, it was found that they could be protected against reinfection in a monocyte-dependent manner [24]. It is suggested that this protective effect is mediated by a BCG-induced increase in innate immune cell function with several distinct features, including higher proinflammatory cytokine responses to unrelated secondary pathogens and activation of the innate immune effector cells such as macrophages and DC. This immune process is mediated by transcriptomic and epigenetic changes in myeloid cells, such as monocytes and macrophages [25]. Monocytes can develop immunological memory, a functional feature widely recognized as innate immune training, to distinguish it from memory in adaptive immune cells. Upon a secondary immune challenge, either homologous or heterologous, trained monocytes/macrophages exhibit a more robust production of proinflammatory cytokines, such as IL-1β, IL-6, and TNF-α, than untrained monocytes [26]. The functional reprogramming of monocytes leads to increased innate immunity-related cytokine and chemokine production that could be induced by the bioactive β-glucans of the fungal cell wall [27].

SARS-CoV-2 infection is closely related to the innate immune system and adaptive immune system. DCs are one of the most important cells in generating immune responses, linking innate immunity and adaptive immunity in viral diseases [28]. DC has also been shown to exhibit immune memory responses [29]. DCs are the most potent professional antigen-presenting cells involved in the antibody production process. In our study, we have shown that *B. subtilis* spores, AM, and CV could stimulate DCs, monocytes, and macrophages to produce IL-1β, IL-6, TNF-α, and other cytokines and chemokines for immunomodulation. The combination of S protein-engineered *B. subtilis,* AM, and CV may produce better outcomes in developing an oral vaccine-like supplement against COVID-19 by boosting the memory response and immune activities of both monocytes and dendritic cells in innate immunity.

Oral vaccinations with *B. subtilis* spores expressing immunogens/antigens for various infectious diseases have been shown to be effective in producing serum IgG and mucosal IgA against the pathogens in pigs [30], mice, and guinea pigs [19,31,32]. Interestingly, the antibody titers in these published studies were quite similar, and only relatively weak antibody responses were elicited (OD < 1.0). The results of our animal studies with *B. subtilis* spores expressing S proteins alone were similar to those published. Oral administration of β-glucan enriched CV and AM extracts enhanced mucosal IgA production and could not be achieved with either CV or AM alone. In our case, the antibody immune responses elicited by oral vaccination with *B. subtilis* spores with or without CV/AM extracts against COVID-19 were mild compared to the conventionally used vaccines such as CoronaVac with intramuscular injection. When mice were fed with S spores prior to CoronaVac vaccination, faster and stronger IgG responses to S protein were observed. The immunogenicity of the S spores with or without AM+CV alone may not be strong enough to induce a systemic IgG immune response against the S protein. However, the mucosal immune response induced by S spores could produce a faster and stronger neutralizing IgG response against the S protein prior to vaccination with the commercial COVID-19 vaccine CoronaVac, thereby producing stronger immunogenicity and IgG response against the ancestral SARS-CoV-2 coronavirus.

With the increasing popularity of probiotics for health promotion, oral probiotic vaccination should be a means to reach those groups. Probiotics have great potential to be incorporated into oral vaccine delivery systems, which may facilitate the induction of mucosal immunity without latent risks of pathogenicity [33]. In addition to AM/CV, β-glucan-enriched yeasts with immunostimulatory properties have recently been tested as a vaccine vehicle and adjuvant. An extended-release vaccine delivery system (GP-diABZI-RBD) consisting of the original SARS-CoV-2 WA1 strain RBD as the antigen and diABZI STING agonist in conjunction with yeast β-glucan particles (GP-diABZI) has been tested [34]. The respiratory and gastrointestinal tracts are the main targets of COVID-19 infection, with 12 to 61% of patients reported to have various gastrointestinal symptoms such as diarrhea, direct damage to intestinal mucosal epithelial cells, malnutrition, and intestinal flora disorders [35]. The mucosal immunity enhanced by our spores, together with β-glucan CV and AM enriched, may provide additional protection against the gastrointestinal tract’s infection by COVID-19.

In both in vitro and in vivo studies, we have demonstrated that *B. subtilis* S spores with or without AM and CV extracts are non-cytotoxic and safe to use. Moreover, a pilot clinical trial was conducted with 16 participants [9] who received oral *B. subtilis* spores expressing the S protein RBD of SARS-CoV-2 on the spore surface or placebo for three courses on three consecutive days. These participants were vaccinated with either BBIBP-CorV [36] or BNT162b2 [37] prior to the clinical trial. In the placebo group, the neutralizing antibody levels gradually declined, whereas the participants receiving the S protein spores showed an increase in neutralizing antibody levels against SARS-CoV-2. No observable local or systemic adverse effects were reported. Apart from the vaccine-like activities, the innate immune-boosting effects of both *B. subtilis* spores and S spores together with CV and AM may provide protection against various respiratory and intestinal infections. *Bacillus*-based probiotics have been shown to strengthen the intestinal barrier and limit inflammatory responses, which may indeed improve digestive health [38]. Recently, *Bacillus* species have been shown to be a potential source of anti-SARS-CoV-2 major protease inhibitors [39].

## 4. Materials and Methods

### 4.1. Preparation of B. subtilis Spores (S Spores) Expressing the Ancestral SARS-CoV-2 RBD of S Protein

Expression constructs containing a cascade of full-length CotC from *B. subtilis*, linker region (peptide sequence: GGGEAAAKGGG) and the RBD of S protein from SARS-CoV-2 were codon optimized for *B. subtilis,* synthesized by Invitrogen GeneArt gene synthesis, and further cloned into shuttle vector pHT01 for *E. coli* and *B. subtilis*. *E. coli* strain DH5α (NEB) was used for the cloning and transformed into *B. subtilis* strain WB800N (Mo Bi Tec) for protein expression. *B. subtilis* transformants were grown at 37 °C, 200 rpm, until OD600 reached 1.0 in 2× LB supplemented with chloramphenicol (5 µg/mL). The culture was induced with a final concentration of 1 mM IPTG and allowed to induce for another 12 h at 37 °C, 200 rpm. The culture was harvested and centrifuged at 4200 rpm for 15 min. The cell pellet was washed with 1× phosphate-buffered saline (PBS, pH 7.4) and resuspended in a half volume of Difco Sporulation Medium (DSM) (8 g nutrient broth No. 4, 0.1% KCl, 1 mM MgSO_4_, and 10 μM MnCl_2_ in 1 L of distilled water supplemented with 0.5 mM CaCl_2_ and 1 μM FeSO_4_). Cells were grown for 24 h at 37 °C, 200 rpm. Vegetative cells were lysed with lysozyme (0.1 mg/mL) at 37 °C for an hour and harvested with centrifugation at 10,000 rpm for 15 min, followed by washing three times with PBS.

**Figure A1:**
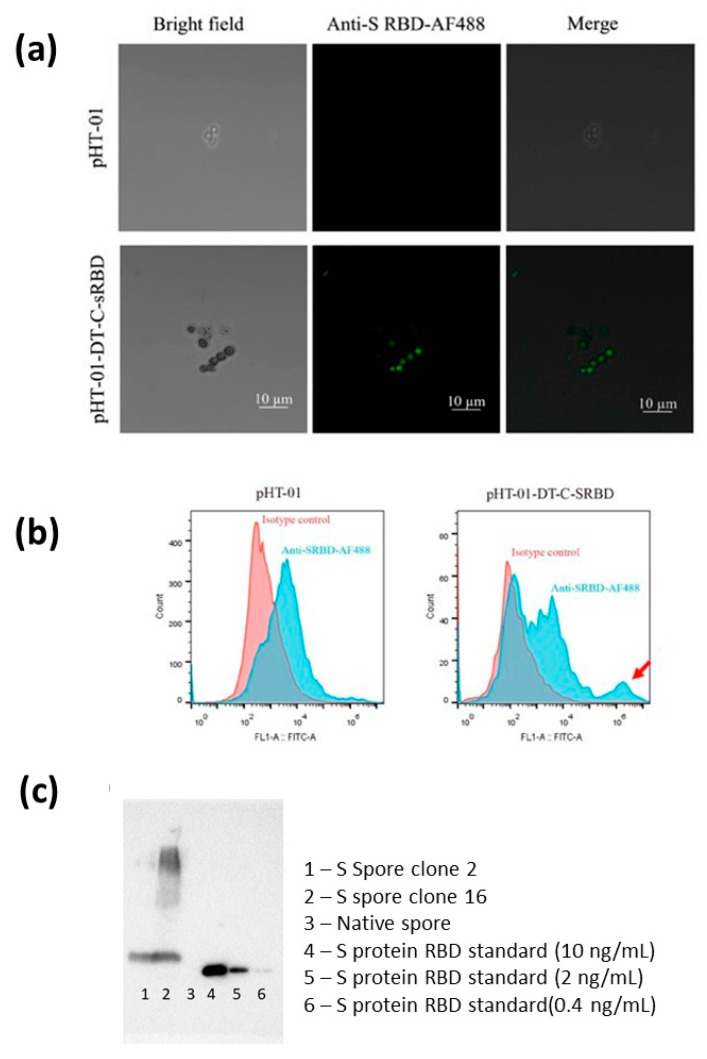
(**a**) Immunofluorescence staining of spike protein on recombinant *B. subtilis* spores visualized by fluorescence microscope. sRBD expression of the ancestral SARS-CoV-2 constructs linked to the coating proteins CotC were cloned and transformed into *B. subtilis* WB800N strain for protein expression. (**b**) Flow cytometry of recombinant *B. subtilis* spores transformed with empty vector pHT-01 and CotC-linked sRBD expression construct. The CotC-linked sRBD-expressing *B. subtilis* spores were immunoreactive against anti-sRBD (indicated by a red arrow). SARS-CoV-2 viral S protein (sRBD) on recombinant *B. subtilis* spores (S spores) from native *B. subtilis* spores (native spores). Immunofluorescence staining and flow cytometry analysis showed that the S protein (sRBD) is expressed on the outer surface of spores. (**c**) The spores were lysed with protein extraction buffer, and the levels of spike protein expressed on the spores were semi-quantitatively quantified by Western blot in comparison with spike protein standards of known concentrations. S proteins from 2 batches were detected (Lane 1 and 2). The amounts of S proteins were 1.7 and 2.4 ng/100 μg, respectively.

To validate the surface expression of the S protein on the spores (S spores), immunofluorescence staining and flow cytometry analysis were performed (Figure A1). The spore was blocked with 5% normal goat serum in PBS for 1 h at room temperature and then stained with rabbit-anti-SARS-CoV-2 Spike RBD antibody (1:500 dilution) (Sino Biological, Beijing, China, Cat. No. 40592-T62) followed by AF488 conjugated donkey anti-rabbit IgG (Invitrogen, Cat. No. R37118, 1:1000). The amount of S protein in the spores was semi-quantified by Western blotting. The spores were lysed with SDS-PAGE buffer and then denatured for 10 min at 100 °C. After centrifugation at 14,800× *g* for 15 min, spore lysate was analyzed in SDS-PAGE and then blotted with monoclonal antibodies against the RBD of S protein. The S protein RBD standard with known concentrations (0.4, 2, and 10 ng/mL) was used for semi-quantitation (Sino Biological, Beijing, China, Cat. No. 40592-V08B). Native spores of non-transformed *B. subtilis* were used as the negative control.

### 4.2. Preparation of the Extract of AM and CV

**Figure A2:**
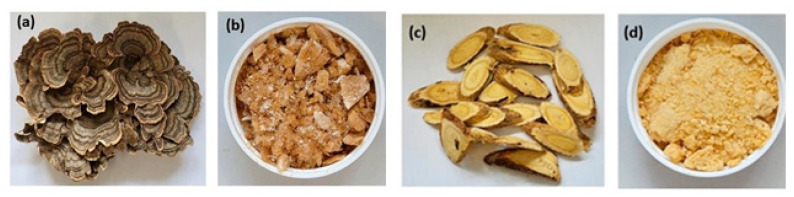
The sample authentication of *Astragalus membranaceus* (Fisch.) Bge (AM) and *Coriolus versicolor* (CV) was performed morphologically and chemically according to the guidelines of the Chinese Pharmacopoeia [40]. The medicinal mushroom CV belongs to the group of Basidiomycetes, in which the fruiting bodies are characterized by hymenium (fertile layer) in vertical pores on the underside of the caps. The upper surface of the cap of CV shows typical concentric zones of different colors, such as brown, white, and grey, with overlapping clusters on dead wood. The cap flat is often triangular or round, with zones of fine hairs. The pore surface is whitish or light brown, with round and twisted pores (**a**). AM was derived from the dried root of *Astragalus membranaceus* (Fisch.) Bge. of the family Fabaceae (Leguminosae). The outer surface was pale brownish-yellow or pale brown with irregular, longitudinal wrinkles or furrows. The texture was hard, tenacious, and uneasily broken. The central part of the old root was occasionally rotten-wood-shaped, blackish-brown, or hollow (**b**). The extraction of AM and CV was performed according to the traditional practice of Chinese medicine preparation. Each of the individual herbs was extracted twice by heating under reflux at 100 °C using 10× distilled water for each extraction. The aqueous extracts for each of the individual herbs were then combined individually and filtered using cotton wool. The filtrates were concentrated under reduced pressure at 60 °C. The concentrated extracts were lyophilized. All the extracts (**c**,**d**) were stored in desiccators at room temperature before use. The extraction efficiency of herbs refers to the number of water extracts extracted from Chinese medicine relative to the number of extracts in the herbs. The yield of extract (extractable components) expressed on a dry weight basis was calculated using the following equation: Yield (g/100 g) = (W1 × 100)/W2 where W1 is the weight of the extract residue obtained after water extraction. W2 is the total weight of the original plant. The extraction efficiencies of CV and AM were 9.85% and 39.27%, respectively.

**Figure A3:**
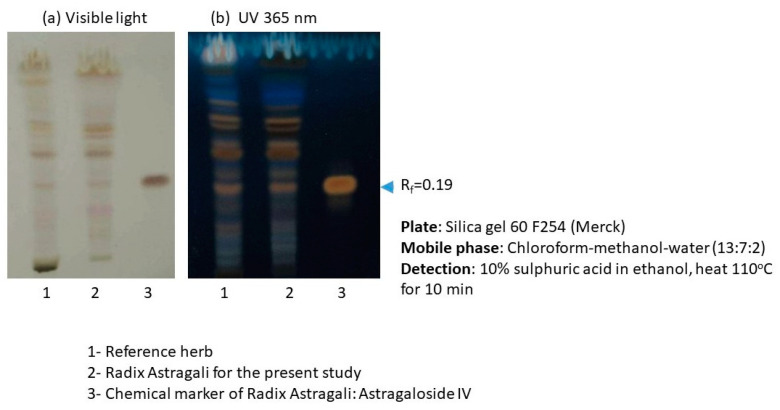
TLC profile of *Astragalus membranaceus* (Fisch.) Bge (AM). Two major chemical markers of RA: astragaloside IV and calycosin 7-*O*-β-d-Glucopyranoside in AM extracts, were further quantified by UPLC-QTOF analysis. The amounts of astragaloside IV and calycosin 7-*O*-β-d-Glucopyranoside were 0.0749% ± 0.0074% and 0.0050% ± 0.0004%, respectively.

**Figure A4:**
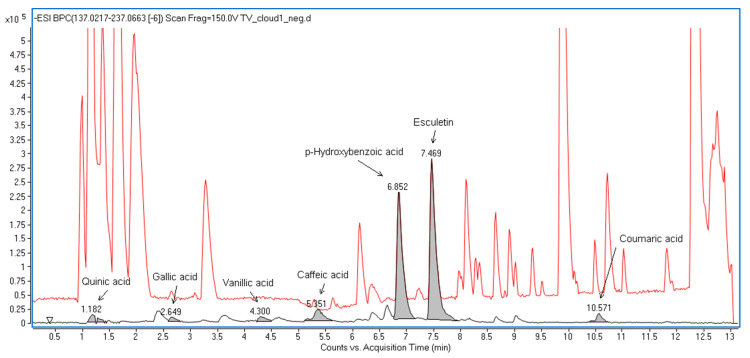
Base peak chromatogram illustrating the tentatively identified compounds in the *Coriolus versicolor* extracts. CV was authenticated by quantification of polysaccharides using the phenol-sulfuric acid colorimetric method. The polysaccharide content in the water extract of CV was 8.02 ± 0.21% (*w*/*w*). The chemical composition of CV was further analyzed by UPLC-QTOF. A total of 7 compounds were tentatively identified in the CV extracts by UPLC-MS. A total of 3 hydroxybenzoic acids, 2 hydroxycinnamic acids, esculetin, and quinic acid were found.

**Figure A5:**
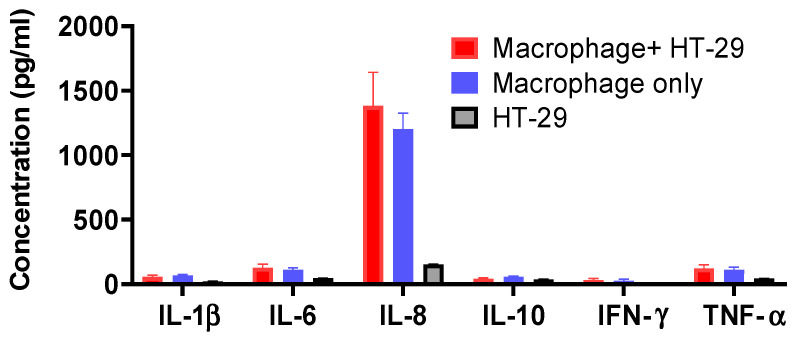
Cytokines production profile (IL-1β, IL-6, IL-8, IL-10, IFN-γ, and TNF-α) of macrophage and HT-29 cell co-culture, macrophage only and HT-29 cells only without treatment.

The dry herbs of AM and CV were purchased from a herbal supplier in Hong Kong (Figure A2). The extraction of AM and CV was performed according to the traditional custom of Chinese medicine preparation. Each of the individual herbs was extracted twice by heating under reflux at 100 °C using 10× distilled water for each extraction. The aqueous extracts for each of the individual herbs were then combined individually and filtered using cotton wool. The filtrates were concentrated under reduced pressure at 60 °C. The concentrated extracts were lyophilized. AM was authenticated using thin-layer chromatography and compared with corresponding chemical markers in accordance with the Chinese Pharmacopoeia [40] (Figure A3). CV was authenticated by quantification of polysaccharides using the phenol-sulfuric acid colorimetric method. The polysaccharide content in CV water extract was 8.02 ± 0.21% (*w*/*w*). The chemical composition of CV was further analyzed with UPLC-QTOF analysis (Figure A4). A total of 7 compounds were tentatively identified in the CV extracts by UPLC-MS. A total of 3 hydroxybenzoic acids, 2 hydroxycinnamic acids, esculetin, and quinic acid were found. The number of endotoxins present in herbal extracts was quantified with a HEK-Blue™ LPS Detection Kit 2, InvivoGen, Toulouse, France). The endotoxin levels of AM and CV were 20.1 and 19.2 EU/mL, respectively. As a small amount of endotoxins was present in the herbal extracts, the antibiotic polymyxin B sulfate (10 μg/μL) [41] was used in the in vitro studies to neutralize the effect of endotoxins in the herbal extracts.

### 4.3. 2.3 3-[4,5-Dimethylthiazol-2-yl]-2,5 Diphenyl Tetrazolium Bromide (MTT) Assay

An in vitro cell toxicity test of the tested compounds on primary human PBMC, purified from human buffy coat, was performed using the MTT assay. Cells were treated with AM, CV (0.1 to 10 mg/mL), native, or S spores (5 to 500 μg/mL) for 48 h. MTT (5 μg/mL) in phosphate-buffered saline (PBS) was then added to each well, and the plates were further incubated for 2 h at 37 °C. The supernatant was removed, and 100 μL of dimethyl sulfoxide (DMSO) was added to each well to dissolve the purple formazan crystals. Absorbance at a wavelength of 540 nm was measured using a microplate reader. Results were expressed as the percentage of untreated cells.

### 4.4. Co-Culture of Human Monocyte Derived Macrophages with Intestinal HT-29 Cells and Monocyte Derived Dendritic Cells (DC) Culture

The innate immunity-boosting effects of native and S spores (0.2–20 μg/mL), AM, and CV water extracts (20–2000 μg/mL) were evaluated. For the development of the oral vaccine-like supplement, an in vitro co-culture system of human monocytes and intestine HT-29 cells was employed to mimic the mucosal area of the intestine. Human monocytes were prepared from fresh human buffy coat obtained from healthy volunteers at the Hong Kong Red Cross Blood Transfusion Service. Peripheral blood mononuclear cells (PBMC) were isolated with Ficoll density (1.082 g/mL) centrifugation for 25 min at 1800 rpm. After RBC lysis, CD14-specific MACS beads (Miltenyi Biotec, Gaithersburg, MD, USA) were used for the enrichment of CD14+ monocytes. To induce macrophage differentiation, CD14+ monocytes were cultured in tissue-culture plates for 6 days in RPMI 1640 medium supplemented with L-glutamine, 10% FCS, 1% Penicillin-Streptomycin, 1% Sodium pyruvate, and 1% Glutamax (GIBCO) and granulocyte macrophage-colony stimulating factor (GM-CSF) (25 ng/mL) at a density of 1.5 × 10^5^/cm^2^. Human intestinal epithelial HT-29 cells were grown to confluence in a 24-well culture plate and then rinsed with PBS at 37 °C to prevent cell detachment. Macrophages (5 × 10^5^/mL) with different treatments were then applied to HT-29 cells in the culture plate. The epithelial cell-macrophage co-cultures were incubated with or without the tested agents for a further 18 h. The concentrations of proinflammatory cytokines/chemokines (IL-1β, IL-6, IL-8, IL-12, IFN-γ, and TNF-α) in the culture supernatant or mouse serum were quantitated with the Bio-plex human cytokine/chemokine multiplex assay using the Bio-plex 200 system (Bio-Rad, Hercules, CA, USA).

For monocyte-derived DC, monocytes were plated at 2 × 10^6^ per mL per well in a 24-well plate and allowed to adhere for 45 min at 37 °C and 5% CO_2_. Non-adherent cells were removed by washing the wells two to three times with a gentle stream of medium. Monocytes were then cultured in the presence of two cytokines: GM-CSF (50 ng/mL) and IL-4 (40 ng/mL) at 37 °C under 5% CO_2_. On day 3, 50% of the medium was replaced with fresh medium and cytokines. The DCs were harvested and washed on day 6. Cell maturation was induced by the tested agents for 48 h. Supernatants from DC cultures were collected after cell harvest and stored at −80 °C until assayed for cytokines. The levels of IL-1β, IL-12, IL-10, TNF-α, and IFN-γ were measured using a multiplex assay.

### 4.5. Immunization Regimens

**Figure 9:**
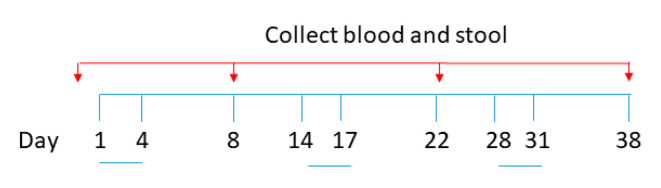
Schematic diagram for oral vaccine-like supplement administration and sample collection.

The experimental procedures were reviewed and approved by the Animal Experimentation Ethics Committee of the Chinese University of Hong Kong (Ref. no. 20-280-ITF). Pathogen-free BALB/c mice (aged 6–8 weeks, 15–20 g body weight) were obtained from the Laboratory Animal Services Centre, the Chinese University of Hong Kong (CUHK). All mice in this study were maintained and handled according to the CUHK Animal Experimentation Ethics Committee Guide for the Care and Use of Laboratory Animals. A total of 25 female mice were divided into 5 groups and orally administered with (1) PBS, (2) 1.0 × 10^9^ S spores, (3) 1.0 × 10^9^ S spores + CV (1.38 g/kg), and AM (0.74 g/kg), (4) AM (0.74 g/kg) and CV (1.38 g/kg) only, and (5) 1.0 × 10^9^ native spore. Suspensions (0.5 mL aliquots) containing approximately 1 × 10^9^ spores were administrated with a stainless-steel round-tip gavage cannula on days 1–4, 14–17, and 28–31 (Figure 9). Blood and fecal samples were collected 3 days before the immunization regimen and on days 8, 22, and 38. At the terminal stage, sera and tissues from the mice, including liver, lung, small and large intestine, were collected. The tissues were perfused with 1 mL of 10% neutral-buffered formalin. The tissues were dehydrated and embedded in paraffin, sectioned, and stained with hematoxylin and eosin. The stained sections were examined with light microscopy to assess the histopathological changes. To check for any toxic effect on the liver function, the sera of the mice were used to measure concentrations of the liver enzyme aspartate aminotransferase (AST) using an AST Activity Assay Kit from Abcam. Individual sera and fecal samples from each group of mice were tested for antibody response. The collected fecal materials were first lyophilized and stored at −20 °C until use. Four fecal pellets per mouse were homogenized in 200 μL of PBS containing 1% BSA and protease inhibitors. The suspensions were centrifuged (16,000× *g*, 10 min, 4 °C), and supernatants were collected. The activities of IgM, IgA, and IgG against the spike protein were measured using ELISA coated with the recombinant SARS-CoV-2 Spike RBD-His Tag protein (Sino Biological, Beijing, China, catalog number: 40592-V08B).

### 4.6. Adjuvant Effects of B. subtilis Spores, AM, and CV on CoronaVac Vaccine

**Figure 10:**
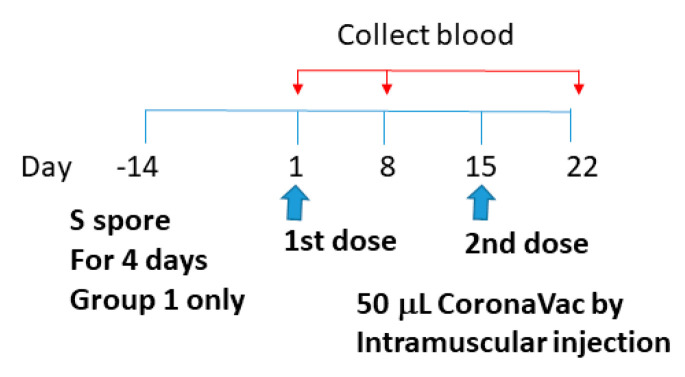
Schematic diagram of the vaccine administration and serum collection for studying the adjuvant effects of *B. subtilis* spores, AM, and CV on the CoronaVac vaccine.

CoronaVac (Sinovac Life Sciences, Beijing, China) was obtained from the Centre for Health Protection, Department of Health, Hong Kong Special Administrative Region. It is an inactivated vaccine containing inactivated SARS-CoV-2 [42,43,44]. Whole viral proteins of SARS-CoV-2 were used for the vaccine, and antibodies against different regions of the viral particle (e.g., nucleocapsid (N) and spike (S) proteins) can be induced by the CoronaVac vaccination. To study the adjuvant effects of *B. subtilis* spores, AM, and CV on the CoronaVac vaccine, sixteen 6-week-old female BALB/c mice were divided into 4 groups: (1) orally fed with S spores (4-day course) 14 days prior to CoronaVac vaccination (50 μL) on day 1 and 15; (2) intramuscularly immunized with CoronaVac only; (3) intramuscularly immunized with CoronaVac (50 μL) and fed with AM (0.74 g/kg) and CV (1.38 g/kg), (days 1–4 and days 14–17); and (4) immunized intramuscularly with PBS (Figure 10). Individual sera and samples of each mice group were tested for antibody response such as IgM, IgA, and IgG against N and S proteins, as described in Section 2.5.

### 4.7. Neutralization Assay

The sera from the CoronaVac vaccinated mice were further tested on the neutralizing antibodies activities against ancestral SARS-CoV-2 using a commercially available SARS-CoV-2 surrogate Neutralizing Antibody ELISA Kit (Thermo Fisher Scientific Inc., Waltham, MA, USA). The 96-well plate was coated with a SARS-CoV-2 Receptor Binding Domain (RBD) antigen. Samples with neutralizing antibodies competed with excessive amounts of biotinylated angiotensin-converting enzyme 2 (ACE2). ACE2 that binds to the RBD would produce signals which were inversely proportional to the level of neutralizing antibodies. The neutralizing antibody control standard included in the kit was used as a positive control. Neutralization (%) for unknown samples ≥20% was counted as positive and <20% was negative. The neutralization (%) for unknown samples was calculated by the following formula:

Neutralization (%) = [1 − (Absorbance of unknown sample at 450 nm/Absorbance of negative control at 450 nm)] × 100(1)

### 4.8. Statistical Analyses

Statistical analyses and significance, as measured by the Student’s *t*-test for paired samples or one-way analysis of variance (ANOVA), were performed using GraphPad PRISM software version 5.0 (GraphPad Software, San Diego, CA, USA). In all comparisons, *p* < 0.05 was considered statistically significant.

## 5. Conclusions

In conclusion, the combination of *B. subtilis* S spores with CV and AM may be useful as a vaccine-like supplement in COVID-19 vaccination. As with other vaccines, prolonged use of oral vaccine-like supplements may induce oral tolerance, and it is not easy to control the immune period [33,45]. Further studies, such as the in vivo functional activities of the macrophages and DC, are needed to clarify these issues before practical use. To enhance the immune response of S spores, increasing the expressions of S proteins in *B. subtilis* spores or adding multivalent SARS-CoV-2 variants S proteins [46,47] to the spores may further enhance the efficacy of the supplement against the current COVID-19 pandemic.

## 6. Patents

The findings from this project have been filed with Chinese (patent no. 202111143384.9) and Hong Kong (patent no. 32021042343.2) patents.

## Data Availability

The data presented in this study are available on request from the corresponding author.

## Sample Availability

The water extracts of *Astragalus membranaceus* (Fisch.) Bge (AM) and *Coriolus versicolor* (CV) are available from the authors.
